# Draft genome sequence of *Salmonella enterica* subsp. enterica serovar Typhimurium SBI_BS16_ABA_BD isolated from broiler chicken in Dhaka, Bangladesh

**DOI:** 10.1128/mra.01175-25

**Published:** 2025-11-25

**Authors:** Anjala Barua Anne, Tamanna Hossen, Md. Safayat Sahib, Raiyan Ul Hakim, Md. Raihan Islam, Sanjana Mahbub Supty, Tarif Md. Faiyaz Saadat, Khondoker Tanjim Islam, Rafid Nahian Rubaiyat, Md. Fakruddin, Spencer Mark Mondol, S. M. Bakhtiar UL Islam

**Affiliations:** 1 Department of Biochemistry and Microbiology, School of Health & Life Sciences, North South University 54495, Dhaka, Bangladesh; 2 Department of Microbiology, University of Dhaka 95324, Dhaka, Bangladesh; Fluxus Inc., Sunnyvale, California, USA

**Keywords:** Bangladesh, BV-BRC, broiler chicken, genome, PGAP, *Salmonella enterica*

## Abstract

Herein, we report the genome of *Salmonella enterica* strain SBI_BS16_ABA_BD, isolated from broiler chicken in Bashundhara, Dhaka, Bangladesh. The 4,990,699-bp genome (51.01% G + C) comprises 57 contigs with 217× coverage with 4,705 coding sequences, 48 tRNA genes, and 3 rRNA genes showing 100% completeness, providing insights into pathogenicity and antimicrobial resistance.

## ANNOUNCEMENT

*S. enterica* subsp. enterica serovar Typhimurium is a Gram-negative, rod-shaped, facultatively anaerobic bacterium (1) and an important pathogen associated with broiler chickens (2). It is a leading cause of foodborne salmonellosis, with contaminated poultry products frequently linked to zoonotic transmission to humans (3). The increasing prevalence of multidrug-resistant strains highlights the need for genomic surveillance of broiler chicken-derived isolates.

*S. enterica* serovar Typhimurium SBI_BS16_ABA_BD strain was isolated from processed broiler chicken cloacal samples sourced from local markets of Aziz Shorok (23.8102717°N, 90.4195257°E), Bashundhara, Dhaka, Bangladesh, in 2024. Cloacal swabs were collected aseptically in Falcon tubes containing buffered peptone water for pre-enrichment and transported to the Microbiology laboratory of North South University. After 10-fold serial dilutions, pure cultures were isolated on selective Salmonella–Shigella agar. Presumptive identification and antibiotic sensitivity testing of six *Salmonella* isolates were performed through Gram staining, biochemical characterization (catalase, oxidase, triple sugar iron, Simmons citrate, methyl red and Voges-Proskauer, motility indole urease, and nitrate reduction assays), and the Kirby-Bauer disk diffusion method. Among these, *S. enterica* strain SBI_BS16_ABA_BD exhibited extensive multidrug resistance and was therefore selected for whole genome sequencing to determine precise serovar identity, antimicrobial resistance and virulence gene profiles, and genomic features relevant to epidemiological surveillance and public health monitoring.

*S. enterica* strain SBI_BS16_ABA_BD was cultured overnight on nutrient agar plates at 37°C before being submitted for whole-genome sequencing at the Genome Sequencing Laboratory, BCSIR, Dhaka, Bangladesh. Genomic DNA was extracted using the Qiagen DNeasy Blood & Tissue Kit (Hilden, Germany), and library preparation was performed with the Nextera DNA Flex Library Preparation Kit (Illumina, San Diego, USA). Whole-genome sequencing was carried out on the Illumina NextSeq 550 platform, generating paired-end raw reads (2 × 150 bp), as previously described in the draft genome sequence announcement of *Bacillus cereus* SBI_SS06_AR_BD (4).

Genome assembly and quality evaluation were carried out using workflows provided by the Bacterial and Viral Bioinformatics Resource Center (BV-BRC) with default parameters. For quality control, Trim Galore version 0.6.5dev (5) was utilized for trimming of paired-end reads (6), and *de novo* assembly was carried out using Unicycler v0.4.8 (7) as adjusted in the BV-BRC pipeline version 3.46.3 (8). The sample contained 3,652,140 reads (261.73 million bases) with an average read length of 35.8. Annotation was carried out using the NCBI Prokaryotic Genome Annotation Pipeline (PGAP) v6.10 (9) under default settings. The assembled genome measured 4,990,699 bp in length, with an average coverage depth of 217×, a G + C content of 51.01%, and an N50 of 147,372 bp. Quality metrics indicated high reliability, with coarse consistency of 99.9%, fine consistency of 98.7%, contamination of 1.20%, and CheckM completeness of 100%. The assembly generated 57 contigs, with 11 ncRNAs and 2 CRISPR arrays. PGAP annotation of *S. enterica* SBI_BS16_ABA_BD identified 4,705 coding sequences, 48 tRNA genes, and 3 rRNA genes.

## Data Availability

The data described in this report are publicly accessible through NCBI BioProject PRJNA1313638 and BioSample SAMN50904862. The draft genome sequences have been deposited in GenBank under the SRA accession number SRR35769298 and the GenBank assembly accession GCA_053044965.1. This Whole Genome Shotgun project has also been deposited at DDBJ/ENA/GenBank under the accession JBROFB000000000 and the version described in this paper is JBROFB010000000.1.
